# Digitally recorded and remotely classified lung auscultation compared with conventional stethoscope classifications among children aged 1–59 months enrolled in the Pneumonia Etiology Research for Child Health (PERCH) case–control study

**DOI:** 10.1136/bmjresp-2021-001144

**Published:** 2022-05-16

**Authors:** Daniel E Park, Nora L Watson, Christopher Focht, Daniel Feikin, Laura L Hammitt, W Abdullah Brooks, Stephen R C Howie, Karen L Kotloff, Orin S Levine, Shabir A Madhi, David R Murdoch, Katherine L O'Brien, J Anthony G Scott, Donald M Thea, Tussanee Amorninthapichet, Juliet Awori, Charatdao Bunthi, Bernard Ebruke, Mounya Elhilali, Melissa Higdon, Lokman Hossain, Yasmin Jahan, David P Moore, Justin Mulindwa, Lawrence Mwananyanda, Sathapana Naorat, Christine Prosperi, Somsak Thamthitiwat, Charl Verwey, Kathleen A Jablonski, Melinda C Power, Heather A Young, Maria Deloria Knoll, Eric D McCollum

**Affiliations:** 1 Department of Environmental and Occupational Health, The George Washington University, Washington, District of Columbia, USA; 2 The Emmes Corporation, Rockville, Maryland, USA; 3 Department of International Health, Johns Hopkins University International Vaccine Access Center, Baltimore, Maryland, USA; 4 Kenya Medical Research Institute - Wellcome Trust Research Programme, Kilifi, Kenya; 5 International Centre for Diarrhoeal Disease Research Bangladesh, Dhaka and Matlab, Bangladesh; 6 Johns Hopkins University Bloomberg School of Public Health, Baltimore, Maryland, USA; 7 Medical Research Council Unit, Basse, Gambia; 8 Department of Paediatrics, The University of Auckland, Auckland, New Zealand; 9 Department of Pediatrics, University of Maryland Center for Vaccine Development, Baltimore, Maryland, USA; 10 Bill & Melinda Gates Foundation, Seattle, Washington, USA; 11 South African Medical Research Council Vaccines and Infectious Diseases Analytics Research Unit, University of the Witwatersrand, Johannesburg, Gauteng, South Africa; 12 Department of Science and Innovation/National Research Foundation: Vaccine Preventable Diseases Unit, University of the Witwatersrand, Johannesburg, Gauteng, South Africa; 13 Department of Pathology and Biomedical Science, University of Otago, Christchurch, New Zealand; 14 Microbiology Unit, Canterbury Health Laboratories, Christchurch, New Zealand; 15 Department of Infectious Disease Epidemiology, London School of Hygiene & Tropical Medicine, London, UK; 16 Department of Global Health, Boston University School of Public Health, Boston, Massachusetts, USA; 17 Sakaeo Crown Prince Hospital, Royal Thai Government Ministry of Public Health, Sakaeo, Thailand; 18 Division of Global Health Protection, Thailand Ministry of Public Health – US CDC Collaboration, Royal Thai Government Ministry of Public Health, Bangkok, Thailand; 19 International Foundation Against Infectious Disease in Nigeria, Abuja, Nigeria; 20 Department of Electrical and Computer Engineering, Johns Hopkins University, Baltimore, Maryland, USA; 21 South African Medical Research Council Vaccines and Infectious Diseases Analytics Research Unit, University of the Witwatersrand, Johannesburg, South Africa; 22 Department of Paediatrics and Child Health, Faculty of Health Sciences, University of the Witwatersrand, Johannesburg, South Africa; 23 Department of Paediatrics and Child Health, University Teaching Hospital, Lusaka, Zambia; 24 Right to Care - Zambia, Lusaka, Zambia; 25 RTI International, Research Triangle Park, North Carolina, USA; 26 Division of Global Health Protection, Thailand Ministry of Public Health – US CDC Collaboration, Royal Thai Government Ministry of Public Health, Nonthaburi, Thailand; 27 The George Washington University Biostatistics Center, Rockville, Maryland, USA; 28 Department of Epidemiology, The George Washington University, Washington, District of Columbia, USA; 29 Global Program in Respiratory Sciences, Eudowood Division of Pediatric Respiratory Sciences, Johns Hopkins School of Medicine, Baltimore, Maryland, USA; 30 Department of International Health, Johns Hopkins University Bloomberg School of Public Health, Baltimore, Maryland, USA

**Keywords:** pneumonia, paediatric lung disaese, respiratory infection

## Abstract

**Background:**

Diagnosis of pneumonia remains challenging. Digitally recorded and remote human classified lung sounds may offer benefits beyond conventional auscultation, but it is unclear whether classifications differ between the two approaches. We evaluated concordance between digital and conventional auscultation.

**Methods:**

We collected digitally recorded lung sounds, conventional auscultation classifications and clinical measures and samples from children with pneumonia (cases) in low-income and middle-income countries. Physicians remotely classified recordings as crackles, wheeze or uninterpretable. Conventional and digital auscultation concordance was evaluated among 383 pneumonia cases with concurrently (within 2 hours) collected conventional and digital auscultation classifications using prevalence-adjusted bias-adjusted kappa (PABAK). Using an expanded set of 737 cases that also incorporated the non-concurrently collected assessments, we evaluated whether associations between auscultation classifications and clinical or aetiological findings differed between conventional or digital auscultation using χ^2^ tests and logistic regression adjusted for age, sex and site.

**Results:**

Conventional and digital auscultation concordance was moderate for classifying crackles and/or wheeze versus neither crackles nor wheeze (PABAK=0.50), and fair for crackles-only versus not crackles-only (PABAK=0.30) and any wheeze versus no wheeze (PABAK=0.27). Crackles were more common on conventional auscultation, whereas wheeze was more frequent on digital auscultation. Compared with neither crackles nor wheeze, crackles-only on both conventional and digital auscultation was associated with abnormal chest radiographs (adjusted OR (aOR)=1.53, 95% CI 0.99 to 2.36; aOR=2.09, 95% CI 1.19 to 3.68, respectively); any wheeze was inversely associated with C-reactive protein >40 mg/L using conventional auscultation (aOR=0.50, 95% CI 0.27 to 0.92) and with very severe pneumonia using digital auscultation (aOR=0.67, 95% CI 0.46 to 0.97). Crackles-only on digital auscultation was associated with mortality compared with any wheeze (aOR=2.70, 95% CI 1.12 to 6.25).

**Conclusions:**

Conventional auscultation and remotely-classified digital auscultation displayed moderate concordance for presence/absence of wheeze and crackles among cases. Conventional and digital auscultation may provide different classification patterns, but wheeze was associated with decreased clinical severity on both.

**WHAT IS ALREADY KNOWN ON THIS TOPIC**

- Digital stethoscopes offer promise in improving the diagnostic capabilities of conventional auscultation using an analogue stethoscope, but there is limited understanding of concordance and associations with clinical outcomes between conventional and digital auscultation among children with pneumonia.

**WHAT THIS STUDY ADDS**

- Among children with pneumonia, conventional auscultation and digital auscultation display moderate concordance, and both demonstrate an association between wheeze and decreased clinical severity.

**HOW THIS STUDY MIGHT AFFECT RESEARCH, PRACTICE AND/OR POLICY**

- Digital stethoscopes have potential for use in research settings and in telemedicine, particularly in low-resource settings where trained auscultation may not be available and where burden of disease is greatest. With further research, detection of wheeze on digital auscultation may inform case management and offer opportunities for reducing unnecessary antimicrobial use.

## Introduction

Despite progress in reducing infant pneumonia mortality, pneumonia remains the leading infectious cause of death globally in children under 5 years of age.^1 2^ Diagnosis of pneumonia is challenging, particularly in low-resource settings where point-of-care diagnostic devices and chest radiographs may not be readily available. Digital stethoscopes offer promise in improving the diagnostic capabilities of conventional auscultation using an analogue stethoscope. Respiratory sounds can be amplified, adjusted and filtered to reduce ambient noise; recorded and shared; and even decomposed into acoustic characteristics and classified through computer-automated algorithms.^3 4^ Additionally, there are promising opportunities for using digital stethoscopes in low-resource settings or telemedicine, as recordings can be sent to experienced clinicians, or automated classification algorithms may aid real-time diagnoses.

Expanding the use of digital auscultation requires a better understanding of how digital auscultation differs from conventional auscultation with an analogue stethoscope. Concordance between conventional auscultation at point-of-care and digital auscultation evaluated by a listening panel or other form of remote evaluation can be impacted by visual observation of the patient and knowledge of the patient’s other clinical signs and symptoms. Furthermore, it is important to understand whether differences in auscultation characteristics across the two approaches affect associations between lung sounds and disease severity or clinical outcomes, as auscultation classifications are often used in clinical decision-making. A better understanding of concordance, predictors of concordance and differences in associations of auscultation with clinical outcomes between conventional and digital auscultation could inform the expanded use of digital auscultation for remote diagnostics in low-resource settings.

We address these knowledge gaps using data from the Pneumonia Etiology Research for Child Health (PERCH) project, a multisite clinical study spanning seven countries.^5^ In order to understand concordance and patterns of auscultatory classifications among children with pneumonia (cases) with distinct clinical and aetiological characteristics, we used a subset of 383 pneumonia cases from the PERCH digital auscultation substudy who had interpretable paired conventional auscultation classifications and concurrently (within 2 hours) recorded digital lung sounds that were classified by an expert listening panel. Furthermore, we evaluated whether associations between auscultation classifications and clinical outcomes differed depending on use of conventional or digital methods using an expanded set of 737 substudy cases with concurrent and non-concurrent paired conventional and digital auscultation classifications. We also considered sensitivity and specificity characteristics of identifying case status using lung classifications among the 737 substudy cases with paired conventional and digital auscultation classifications and 284 community controls who had interpretable digital auscultation recordings only.

## Methods

### Overview of PERCH

Between August 2011 and January 2014, the PERCH project enrolled children in nine locations in seven countries (Basse, The Gambia; Bamako, Mali; Lusaka, Zambia; Soweto, South Africa; Kilifi, Kenya; Dhaka and Matlab, Bangladesh; and Nakhon Phanom and Sa Kaeo, Thailand). Study design and methods have been previously described.^6 7^ In brief, PERCH enrolled hospitalised severe and very-severe pneumonia cases defined as children with cough or difficulty breathing and with either lower chest wall in-drawing or signs of WHO-defined very severe pneumonia at presentation to the hospital. Cases eligible for PERCH resided inside the study catchment area and could not have been hospitalised in the 14 days prior to the current admission for any reason or discharged from the hospital for a pneumonia admission within 30 days prior. Cases with wheeze were excluded if case-defining lower chest wall in-drawing resolved after bronchodilator therapy. PERCH controls were children without case-defining pneumonia randomly selected from the same catchment area as cases, and frequency matched to cases by age group (1–5 months, 6–11 months, 12–23 months and 24–59 months). Controls were eligible for inclusion regardless of presence of respiratory symptoms, unless study staff determined that the child met the case definition for pneumonia.

### Conventional and digital auscultation

The digital auscultation substudy was nested within the PERCH study and was conducted at all sites except for Mali. The digital auscultation substudy was a convenience subset of cases and controls which began during the second half of PERCH enrolment; there were no additional criteria for enrolment.

Providers used their own conventional stethoscopes to document standardised auscultation classifications (presence of wheeze and/or crackles) at the time of clinical assessment during enrolment.^8^ Conventional chest auscultation was not done on controls. Providers used commercial digital stethoscopes (ThinkLabs ds32a) to record sounds from pre-specified chest locations. Digital auscultation occurred either at the enrolment clinical assessment, concurrent with the conventional auscultation, or later based on availability of equipment and trained staff. An external microphone affixed to the stethoscope recorded ambient noise. Research staff uploaded de-identified recordings from the sound recorder to study servers. Unwanted ambient noise was removed using a novel automated multiband denoising filter developed and validated by Johns Hopkins University sound engineers and physicians.^9^

A panel of two pediatric-experienced physicians and six paediatricians classified the filtered recordings as wheeze and/or crackles, neither crackles nor wheeze or uninterpretable after training using standardised criteria, as previously described.^10^ Recordings were randomly assigned to two panellists (ie, primary listeners). If the primary listeners disagreed on the lung sound, a third panellist blinded to the prior assessments was randomly selected to interpret the lung sounds. If the third listener’s classification agreed with either of the primary listeners, the classification was considered final. Any remaining discordant recordings were classified by consensus between one panellist and an external paediatric pulmonologist. The listening panel was blinded to all patient information, including case–control status.

Uninterpretable sound files were excluded from all analyses. When evaluating associations between auscultation classifications and clinical characteristics, auscultation classifications were grouped into: (1) any wheeze, (2) crackles-only and (3) neither crackles nor wheeze.

### Covariates and clinical characteristics

In addition to demographic characteristics, we assessed cases for clinical characteristics including severe malnutrition (<−3 Z score weight-for-age), tachypnoea (respiratory rate ≥60 breaths/min <2 months of age, ≥50 breaths/min 2–11 months, ≥40 breaths/min >12 months), malaria parasitaemia (conducted when clinically indicated, or universally in endemic areas: Kenya, The Gambia and Zambia), anaemia (haemoglobin <7.5 g/dL) and hypoxia at admission (oxygen saturation <92%, or <90% for sites at elevation above 1200 m (Zambia and South Africa), or supplemental oxygen use if a room air oxygen saturation reading was not available). It was standard practice to administer supplemental oxygen for all children admitted to hospital with a diagnosis of severe pneumonia at the South African site, and therefore South Africa was excluded from evaluations using supplemental oxygen as a clinical outcome. Chest X-rays were obtained from cases and interpreted by two PERCH chest radiograph reading panel members using the WHO method, which defined radiographic pneumonia as primary endpoint pneumonia with or without other infiltrate.^11^ We assessed vital status during follow-up visits or telephone interviews conducted 30 days after hospital admission (window of 21–90 days).

### Specimen collection

We collected nasopharyngeal and oropharyngeal (NP-OP) swabs, blood cultures, lung aspirate, pleural fluid and gastric aspirates from cases. Respiratory tract samples were tested using a 33-pathogen multiplex quantitative PCR (FTD Resp-33, Fast Track Diagnostics, Sliema, Malta) and cultures, as previously described.^12–18^ We defined microbiologically confirmed pneumonia cases as children with bacteria isolated from a normally sterile site, including lung aspirate, pleural fluid and blood culture. Likely pneumococcal pneumonia cases were culture-positive for pneumococcus in blood or lung specimens, lung aspirate or pleural fluid PCR-positive or had a combination of chest X-ray consolidation and high pneumococcal DNA load (>2.2 log_10_ copies/uL) in whole blood, and/or consolidation with high-density (>6.9 log_10_ copies/uL) pneumococcus in NP-OP swab samples.

### Statistical analysis

#### Conventional and digital auscultation concordance

We evaluated concordance between conventional and digital auscultation classifications among 383 cases who had conventional auscultation and digital recordings taken concurrently (within 2 hours of each other). The conventional auscultation time was based on the start of the case clinical assessment, while the exact time of the digital auscultation was available. We assessed concordance using Cohen’s kappa statistic and a prevalence-adjusted, listener bias-adjusted Kappa (PABAK).^19^ The Cohen’s kappa statistic is affected by bias between observers and prevalence of the outcome, particularly where the prevalence is low or high; the PABAK provides an additional measure of observer agreement that alleviates the effect of bias and prevalence on kappa agreement. A kappa value of 0–0.20 is considered slight agreement, 0.21–0.40 as fair, 0.41–0.60 as moderate, 0.61–0.80 as substantial and above 0.80 as high.^20^ As a sensitivity analysis, we assessed effect modification by time between conventional and digital auscultation. We calculated overall agreement using the total number of times the classifications agreed, divided by the total number of classifications, along with a Wilson score binomial CI.

#### Conventional and digital auscultation classifications among cases with distinct clinical and aetiological characteristics

Among cases with concurrently collected conventional and digital auscultation (n=383), we used χ^2^ tests to compare distributions of auscultation classifications across groups of children in whom auscultatory classifications were expected to have differing prevalence of crackles and/or wheeze, including children with likely pneumococcal pneumonia, high C-reactive protein (CRP≥40 mg/L), and children who were discharged alive in ≤2 days with a non-colonising virus detected on NP-OP PCR (as a proxy for an acute viral infection).

#### Predictors of concordance between conventional and digital auscultation

Using all 737 substudy cases with both concurrent and non-concurrent paired conventional and digital auscultation classifications available, we further evaluated whether clinical and demographic characteristics were associated with concordance between auscultation methods. We used logistic regression to evaluate independent associations between concordance and age, sex, WHO-defined clinical severity, site, duration between conventional and digital auscultation and crying. Subsequently, we used a multivariate logistic regression model adjusted for all characteristics associated with agreement at the significance level of 0.20, including age and site.

#### Conventional and digital auscultation classifications and associations with disease severity and clinical outcomes

Among all 737 cases with interpretable paired conventional and digital auscultation classifications available, associations between auscultation classification category and disease severity and clinical outcomes were evaluated for conventional and digital auscultation using logistic regression adjusted for age, sex and site. Disease severity and clinical outcomes included high CRP, WHO-classified very severe pneumonia, abnormal chest X-ray findings, discharged alive from hospital admission in ≤2 days and death within 30 days of hospital admission.

#### Digital auscultation classifications among controls and associations of classifications with case–control status

We evaluated the prevalence of crackles and wheeze among 284 community controls with interpretable digital auscultation recordings available. Sensitivity, specificity, negative predictive value and positive predictive value for determining case–control status by using presence of crackles nor wheeze was calculated using all 737 cases and 284 controls from the substudy.

Statistical analyses were conducted in SAS, V.9.4, and R, V.3.3.1.

### Patient and public involvement

Patients or the public were not involved in the design, conduct, reporting or dissemination plans of our research.

## Results

Enrolment of eligible PERCH cases into the digital auscultation substudy ranged from 23.6% and 27.0% in South Africa and The Gambia, respectively, to 85.0%–100% at other sites (online supplemental table S1). Compared with non-enrolled cases, enrolled cases tended to be older, had a higher case fatality ratio, more often had malaria parasitaemia and anaemia and less frequently had hypoxaemia, supplemental oxygen use (ever) and abnormal chest X-rays.

10.1136/bmjresp-2021-001144.supp1 Supplementary data

**Figure 1:**
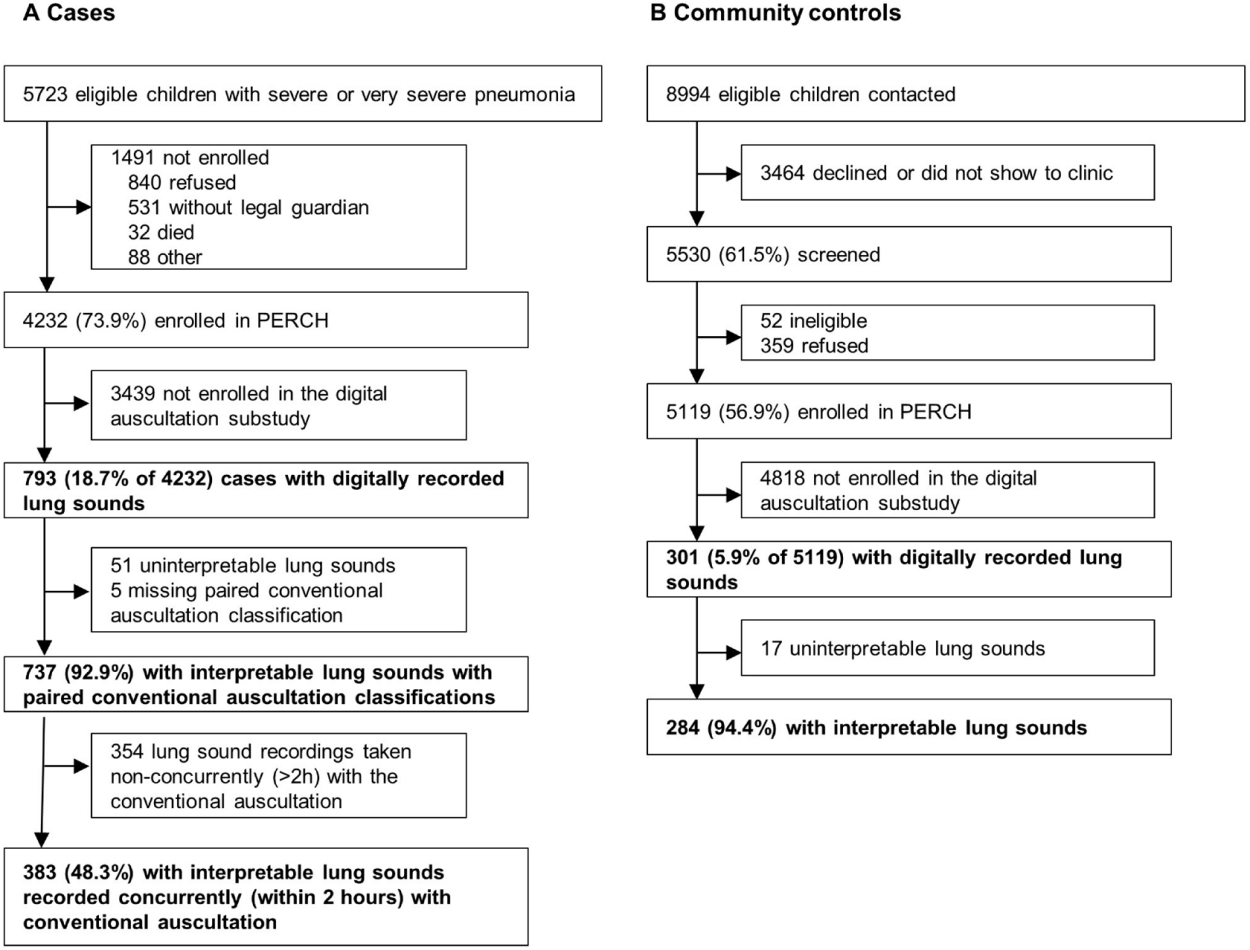
Case (A) and control (B) enrolment into the PERCH digital auscultation substudy (bolded). PERCH, Pneumonia Aetiology Research for Child Health.

Lung sounds from 793 cases and 301 controls were recorded at the study sites, denoised by an automated algorithm, and evaluated by the listening panel (figure 1). Recordings from 51 (6.4%) cases and 17 (5.6%) controls were determined to be uninterpretable by the listening panel, and five cases were missing a conventional auscultation classification. Interpretable paired conventional and digital auscultation classifications were available from 737 cases. Interpretable digital auscultation classifications were available for 284 controls. Among cases with paired conventional and digital auscultation classifications, 383 (52.0%) were conducted concurrently (within 2 hours) of each other (median 25 min), 304 (41.2%) were conducted more than 2 hours apart (median 15.7 hours), and 50 (6.8%) were missing time information.

Among the 737 cases with paired conventional and digital auscultation classifications, most (68.1%) were enrolled from Zambia, Bangladesh and Kenya (table 1). The South Africa and Kenyan sites contributed a smaller proportion of cases with concurrent auscultation (0.3% and 3.7%, respectively) compared with their overall substudy case contribution (12.5% and 16.7%, respectively). Digital auscultation controls were predominantly from Thailand (58.8%), The Gambia (16.2%) and Zambia (12.7%). Among the 737 cases, crackles-only classifications were most common on conventional auscultation (36.6%), followed by 29.0% with neither crackles nor wheeze, 28.0% with both crackles and wheeze and 6.4% with wheeze-only. Using digital auscultation, 38.1% had neither crackles nor wheeze, 27.0% were classified as both crackles and wheeze, 22.7% had wheeze-only and 12.2% had crackles-only (online supplemental table S2).

**Table 1:** Characteristics of children with severe pneumonia and community controls in the digital auscultation substudy

Characteristic	No. (%) with available information		
	Cases		Controls
	All substudy cases	Concurrent auscultation	
Total	737	383	284
Age			
1–5 months	315 (42.7)	153 (40.0)	69 (24.3)
6–11 months	172 (23.3)	102 (26.6)	67 (23.6)
12–23 months	148 (20.1)	78 (20.4)	76 (26.8)
24–56 months	102 (13.8)	50 (13.1)	72 (25.4)
Female sex	315 (42.7)	153 (40.0)	133 (46.8)
Site			
Zambia	234 (31.8)	154 (40.2)	36 (12.7)
Bangladesh	145 (19.7)	121 (31.6)	23 (8.1)
Kenya	123 (16.7)	14 (3.7)	3 (1.1)
South Africa	92 (12.5)	1 (0.3)	9 (3.2)
The Gambia	79 (10.7)	71 (18.5)	46 (16.2)
Thailand	64 (8.7)	22 (5.7)	167 (58.8)
HIV-positive	58 (7.9)	26 (6.8)	2 (0.7)
Malnourished (weight-for-age)	165/694 (23.8)	80/363 (22.0)	18 (6.3)
Clinical			
Very severe pneumonia (WHO definition)	244 (33.1)	93 (24.3)	–
Microbiologically confirmed pneumonia	22/705 (3.1)	11/361 (3.1)	–
Confirmed pneumococcal pneumonia	6/705 (0.9)	4/361 (1.1)	–
Likely pneumococcal pneumonia	53/619 (8.6)		–
Hypoxaemia at admission	245/736 (33.3)	83/382 (21.7)	–
Supplemental oxygen (ever)*	238/644 (37.0)	116/382 (30.4)	–
Tachypnoea	589/726 (81.1)	314/379 (82.9)	–
Malaria parasitaemia	16/722 (2.2)	4/371 (1.1)	–
Anaemia	20 (2.7)	8 (2.1)	–
Chest X-ray conclusion			–
Radiographic pneumonia	167 (22.7)	71 (18.5)	–
Other infiltrate only	138 (18.7)	65 (17.0)	–
Normal	322 (43.7)	195 (50.9)	–
Uninterpretable	65 (8.8)	32 (8.4)	–
Missing	45 (6.1)	20 (5.2)	
Died in hospital or within 30 days of admission	68 (9.2)	32 (8.4)	–
Died in hospital	56 (7.6)	26 (6.8)	–
Died post discharge, within 30 days of admission	12 (1.9)	6 (1.6)	–
Missing 30-day vital status	104 (14.1)	66 (17.2)	–

*Excludes South Africa due to near uniformity of receiving oxygen at South Africa.

### Conventional and digital auscultation concordance

**Figure 2:**
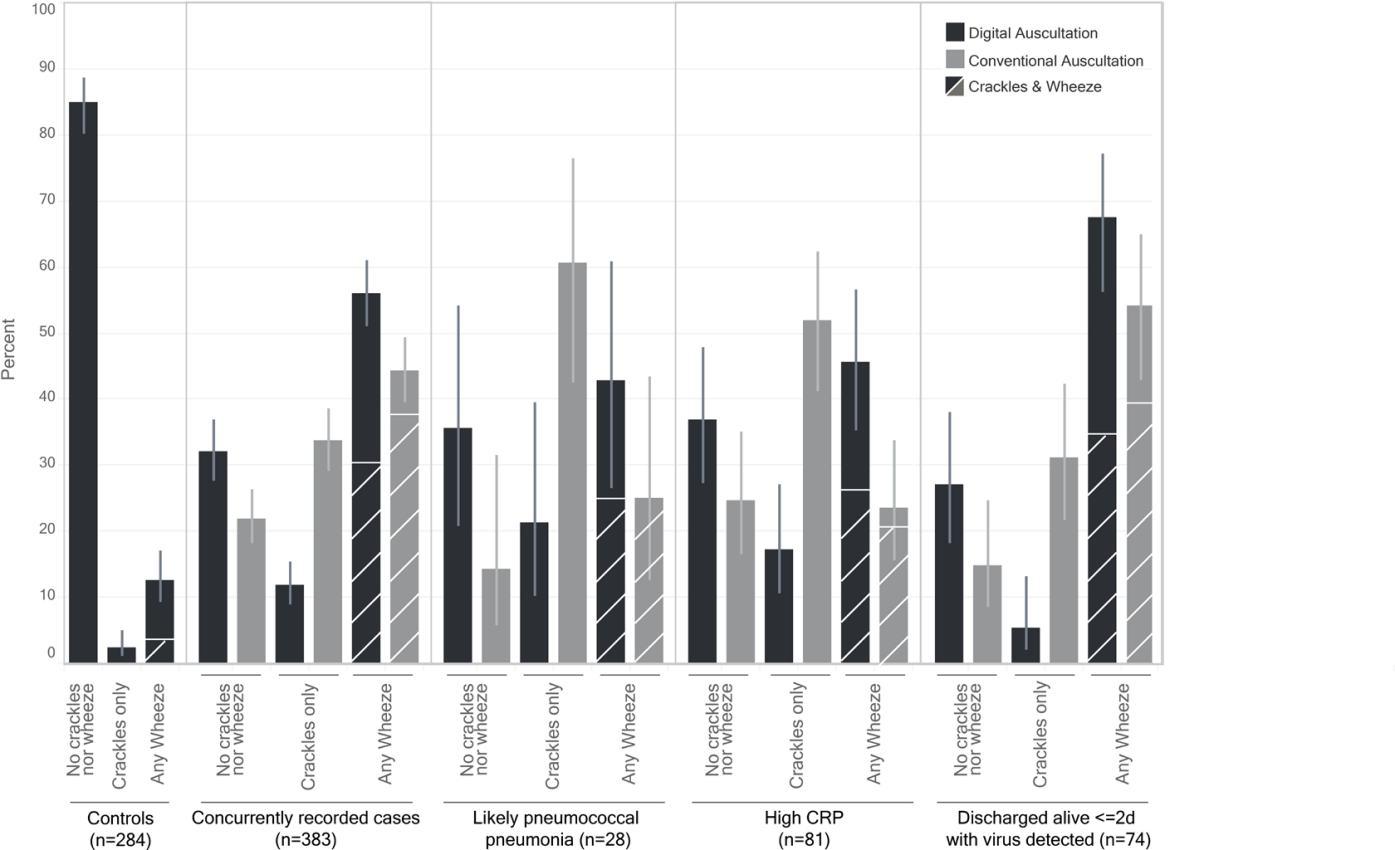
Percent of children with classifications^a^ of neither crackles nor wheeze, crackles only, any wheeze and crackles and wheeze (hatched boxes) on digitally recorded and panel classified auscultation and conventional auscultation, by clinical and aetiological categories. (a) Among all controls with digital recordings and cases with concurrent digital recording and conventional auscultation classifications. Smaller vertical lines indicate Wilson score binomial proportional 95% CIs. CRP, C-reactive protein.

Among 383 children with concurrent conventional and digital auscultation evaluations, there was moderate agreement with classification of any crackles and/or wheeze versus neither crackles nor wheeze (75.2% overall agreement, PABAK=0.50, 95% CI 0.41 to 0.59). Concordance was fair for crackles and wheeze when considered independently (table 2). Digital auscultation was more likely to result in a wheeze-only or neither crackles nor wheeze classification compared with conventional auscultation, whereas the proportion with both crackles and wheeze was similar (figure 2).

**Table 2:** Concordance between digitally recorded and human listener classified lung auscultation and conventional stethoscope classifications, among cases with concurrent digital recording and conventional classification (within 2 hours)

Comparison	N (%)		Agreement on conventional and digital	Overall agreement	Kappa	PABAK
	Digital	Conventional	n	n (%)	Coefficient	Coefficient
Total	383	383				
Any abnormal						
No crackles nor wheeze	123 (32.1)	84 (21.9)	56	288 (75.2)	0.38 (0.28 to 0.48)	0.50 (0.41 to 0.59)
Any crackles or wheeze	260 (67.9)	299 (78.1)	232			
Crackles						
Crackles-only	45 (11.8)	129 (33.7)	20	249 (65.0)	0.07 (−0.02 to 0.15)	0.30 (0.21 to 0.39)
Not crackles-only	338 (88.3)	254 (66.3)	229			
Wheeze						
Any	216 (56.3)	170 (44.3)	123	244 (63.5)	0.28 (0.19 to 0.37)	0.27 (0.18 to 0.37)
None	168 (43.8)	214 (55.7)	121			

Although concordance was similar when comparing shorter and longer duration between conventional and digital auscultation, digital recordings taken >2 hours from the conventional auscultation had a higher proportion of classifications without crackles nor wheeze compared with those <2 hours (44.3% vs 32.0%, χ^2^ p=0.001) (online supplemental table S3).

### Conventional and digital auscultation classifications among cases with distinct clinical and aetiological characteristics

Among 383 children with concurrent digital and conventional auscultation, more children had crackles-only on conventional compared with digital auscultation among 28 likely pneumococcal pneumonia cases (60.7% vs 21.4%, p=0.011). Crackles-only were also more commonly detected on conventional auscultation among 81 cases with CRP ≥40 mg/L (51.9% vs 17.3%, p<0.001), a marker typically associated with bacterial infection (online supplemental table S2). Among children who were discharged alive in ≤2 days with a non-colonising virus detected on NP-OP PCR, conventional auscultation classified 31.1% with crackles-only and 14.9% with wheeze-only; conversely on digital auscultation just 5.4% had crackles-only and 33.8% had wheeze-only (p<0.001).

### Predictors of concordance between conventional and digital auscultation

Disagreement between conventional and digital auscultation in classifying normal and abnormal classifications were similar across sex, WHO-defined pneumonia severity, time delay between conventional and digital auscultation and crying on recording (table 3). Recordings from The Gambia and Bangladesh had the highest rates of agreement (84.8% and 77.2%, respectively) compared with other sites (range 65.9%–69.6%), with The Gambia site being associated with concordance (adjusted OR (aOR)=2.4, 95% CI 1.2 to 4.8, p=0.014). There were no other significant predictors of agreement.

**Table 3:** Predictors of concordance between digitally recorded and human listener classified lung auscultation and conventional stethoscope classifications, comparing auscultation of any crackles and/or wheeze and neither crackles nor wheeze

Characteristic	Total N=737	Agreement n=530	OR			Adjusted OR*		
	n	n (%)	Estimate	95% CI	P value	Estimate	95% CI	P value
Age								
1–5 months	315	233 (74.0)	1.37	0.84 to 2.23	0.208	1.46	0.87 to 2.44	0.152
6–11 months	172	117 (68.0)	1.07	0.63 to 1.81	0.805	1.08	0.62 to 1.86	0.792
12–23 months	148	112 (75.7)	1.75	0.98 to 3.11	0.058	1.75	0.97 to 3.15	0.062
24–56 months	102	68 (66.7)	Ref	–	–	Ref	–	–
Sex								
Male	422	305 (72.3)	Ref	–	–	Ref	–	–
Female	315	225 (71.4)	0.94	0.67 to 1.31	0.703	0.98	0.70 to 1.38	0.919
Severity								
Very severe pneumonia	244	177 (72.5)	1.09	0.77 to 1.55	0.618	–	–	–
Severe pneumonia	493	353 (71.6)	Ref	–	–	–	–	–
Site								
Zambia	234	162 (69.2)	Ref	–	–	Ref	–	–
Bangladesh	145	112 (77.2)	1.49	0.92 to 2.40	0.104	1.49	0.91 to 2.44	0.113
Kenya	123	81 (65.9)	0.85	0.53 to 1.35	0.479	0.94	0.55 to 1.59	0.805
South Africa	92	64 (69.6)	1.16	0.65 to 2.09	0.616	1.25	0.66 to 2.39	0.495
The Gambia	79	67 (84.8)	2.37	1.21 to 4.67	**0.012**	2.40	1.20 to 4.80	**0.014**
Thailand	64	44 (68.8)	0.96	0.53 to 1.75	0.905	1.08	0.57 to 2.04	0.821
Duration between digital and conventional auscultation								
<2 hours	383	288 (75.2)	1.36	0.98 to 1.89	0.060	1.14	0.75 to 1.75	0.541
>2 hours	332	229 (69.0)	Ref	–	–	Ref	–	–
Missing time information	22	13 (59.1)	–	–	–	–	–	–
Intermittent crying on recording								
Yes	330	244 (73.9)	Ref	–	–	Ref	–	–
No	407	286 (70.3)	1.18	0.85 to 1.64	0.337	1.19	0.84 to 1.69	0.333

Bold values denote statistical significance at the p <0.05 level.

*Adjusted for age, sex, site, duration between digital and conventional auscultation and intermittent crying on recording.

### Conventional and digital auscultation classifications and associations with disease severity and clinical outcomes

Among all substudy cases, having crackles-only was generally indicative of higher severity, and wheeze with reduced severity on both digital and conventional auscultation (table 4). In both conventional and digital auscultation, children with crackles-only had the highest proportion with CRP ≥40 mg/L (33.7% and 32.7%, respectively). Compared with neither crackles nor wheeze, any wheeze was associated with lower CRP (<40 mg/L) on conventional auscultation (aOR=0.50, 95% CI 0.27 to 0.92) and was inversely associated with very severe pneumonia status on digital auscultation (aOR=0.67, 95% CI 0.46 to 0.97). Crackles-only classifications were associated with abnormal chest X-ray findings on both conventional and digital auscultation (aOR=1.53, 95% CI 0.99 to 2.36; aOR=2.09, 95% CI 1.19 to 3.68, respectively). Mortality was highest among children without crackles nor wheeze on conventional auscultation (17.1%), and was highest among children with crackles-only on digital auscultation (17.4%). Mortality was lowest among children with any wheeze on both conventional auscultation (3.8%) and digital auscultation (6.4%). Crackles-only classifications were associated with mortality compared with any wheeze classifications on digital auscultation only (aOR=2.70, 95% CI 1.12 to 6.25).

**Table 4:** Clinical characteristics by auscultation classifications using digitally recorded and panel classified auscultation and conventional auscultation

Characteristic	Digital auscultation			Conventional auscultation		
	Proportion with clinical outcome n/N (%)	χ^2^ p value*	aOR (95% CI)	Proportion with clinical outcome n/N (%)	χ^2^ p value*	aOR (95% CI)
Total	**N=737**			**N=737**		
High CRP >40 mg/L, n=148		**0.030**			**<0.001**	
No wheeze, no crackles	60/226 (26.6)		Ref	47/167 (28.1)		Ref
Crackles only	28/83 (33.7)		1.50 (0.85 to 2.63)	74/226 (32.7)		1.25 (0.79 to 1.98)
Any wheeze	60/294 (20.4)		0.79 (0.51 to 1.23)	27/210 (12.9)		0.50 (0.27 to 0.92)
Very severe pneumonia, n=244		**<0.001**			**<0.001**	
No wheeze, no crackles	120/281 (42.7)		Ref	97/214 (45.3)		Ref
Crackles only	30/90 (33.3)		0.75 (0.44 to 1.28)	96/270 (35.6)		0.88 (0.59 to 1.32)
Any wheeze	94/366 (25.7)		0.67 (0.46 to 0.97)	51/253 (20.2)		0.79 (0.47 to 1.31)
Abnormal chest X-ray†, n=305		**0.003**			**<0.001**	
No wheeze, no crackles	111/224 (49.6)		Ref	85/170 (50.0)		Ref
Crackles only	50/76 (65.8)		2.09 (1.19 to 3.68)	134/224 (59.8)		1.53 (0.99 to 2.36)
Any wheeze	144/327 (44.0)		1.05 (0.72 to 1.52)	86/233 (36.9)		0.84 (0.50 to 1.40)
Discharged alive in <2 days, n=203		0.489			0.864	
No wheeze, no crackles	78/280 (27.9)		Ref	55/214 (25.7)		Ref
Crackles only	19/88 (21.6)		0.73 (0.40 to 1.32)	75/269 (27.9)		1.39 (0.90 to 2.16)
Any wheeze	99/362 (27.4)		0.96 (0.65 to 1.41)	66/247 (26.7)		2.01 (1.17 to 3.47)
Died		**0.001**			**<0.001**	
No wheeze, no crackles	35/238 (14.7)		Ref	29/170 (17.1)		Ref
Crackles only	12/69 (17.4)		2.04 (0.88 to 4.70)	30/224 (13.4)		0.79 (0.44 to 1.43)
Any wheeze	21/326 (6.4)		0.76 (0.40 to 1.43)	9/239 (3.8)		0.43 (0.17 to 1.06)

Bold values denote statistical significance at the p <0.05 level.

*Overall unadjusted χ^2^ p values.

†Consolidation with or without other infiltrate

### Digital auscultation classifications among controls and associations of classifications with case–control status

Only 15.1% of the 284 controls had abnormal lung sounds: 2.5% had crackles-only and 12.7% had any wheeze (online supplemental table S2). Notably, clinical symptoms consistent with an acute respiratory infection were reported by 94 (33.1%) controls; among these controls, 17.0% had abnormal lung sounds: 2.1% with crackles-only and 14.9% with any wheeze. Using all cases and controls, the presence of crackles or wheeze to classify pneumonia case status yielded 61.4% sensitivity, 84.9% specificity, 46.2% negative predictive value and 91.4% positive predictive value (online supplemental table S4).

## Discussion

Sequentially collected conventional auscultation classifications and digitally recorded and remotely classified lung auscultation classifications have fair-to-moderate concordance when evaluating crackles and wheeze lung sounds among pneumonia cases with concurrent conventional and digital auscultation. Conventional and digital auscultation may result in different classification patterns, with a higher proportion with crackles on conventional auscultation and a higher proportion with wheeze on digital auscultation. In an expanded sample of pneumonia cases with both concurrent and non-concurrent paired conventional and digital auscultation, patient-level characteristics did not predict concordance. Presence of crackles was generally predictive of greater clinical severity among pneumonia cases, and wheeze was associated with decreased clinical severity.

Despite having been an established and widely used diagnostic tool for centuries, the accuracy and reliability of chest auscultation for pneumonia diagnosis has been questioned, even when using near-simultaneous auscultation with identical equipment. Foundationally, there is no readily available gold standard to assess auscultation accuracy. There are uncertainties regarding variation within lung sounds between breaths of different volume, temporal changes between breaths, intra-provider variability over time and inter-provider variability at the same time point with the same patient.^21^ These uncertainties may be exacerbated in the examination of young children. In a study of Norwegian adults, kappa agreement between providers was κ=0.43 for inspiratory wheezes, κ=0.56 on expiratory wheezes, κ=0.46 on inspiratory crackles and κ=0.20 for expiratory crackles.^22^ In a prospective study of 102 infants, Elphick *et al*^23^ reported κ=0.07 for wheeze and κ=0.36 for crackles between two experienced clinicians.^23^ Melbye *et al*^24^ found comparatively lower agreement among paediatric recordings compared with adult recordings.

Nearly all studies of agreement have been conducted in controlled environments in high-income country settings where the clinical environment is typically quieter than many low-income and middle-income country settings. For example, comparing agreement between conventional and digital auscultation, Kevat *et al*^25^ compared intra-listener (within one provider) concordance from children in a tertiary paediatric facility in Melbourne, Australia, and found moderate concordance for wheeze (κ=0.44 and 0.55) and near-perfect concordance for crackles. Digital auscultatory recording and remote classification presents challenges due to the inability to visually observe the patient, including inspiratory and expiratory phases, clinical picture, and may have external noise, especially in many busy low-income and middle-income settings where PERCH was conducted. Despite these challenges, the concordance levels in our study demonstrate that digitally recorded and remotely classified lung auscultation can achieve results similar to inter-provider concordance using identical equipment in ideal settings.

Patterns of auscultation classifications were different between conventional and digital auscultation, with digital auscultation classifications having a greater proportion of wheeze and a lower proportion of crackles. Acute bronchiolitis is often caused by viruses and is associated with wheeze.^26^ In our likely acute viral infection group, wheeze-only classifications were significantly more common using digital auscultation (online supplemental table S2). Kevat *et al*^25^ reported better sensitivity for detecting wheeze using digital stethoscopes compared with conventional stethoscopes. Conventional bell and diaphragm stethoscopes may attenuate higher frequency sounds such as wheeze, whereas digital stethoscopes can capture sounds across the full range of audible sound frequencies.

Sensitive detection of wheeze may be an informative diagnostic feature. Wheeze on digital auscultation was associated with both lower mortality (compared with crackles) and lower odds of having very severe pneumonia (compared with other sound classifications). Children without crackles nor wheeze may be a mix of children without severe lung involvement, or alternatively may be very severe cases with low lung function and volume and unable to generate crackles or wheeze sounds. We previously reported that wheeze on digital auscultation was associated with a lower odds of radiographic pneumonia compared with children without crackles nor wheeze among children with severe pneumonia but no WHO danger signs.^27^ Future research may explore whether this common but less-severe case group may benefit if digital auscultation adds differential diagnostic capacity with regards to severity or aetiology to help guide appropriate triage and antibiotic prescribing.^27^

Crackles were detected less frequently on digital auscultation compared with conventional auscultation in our study. Crackles were associated with abnormal chest radiography using both digital and conventional auscultation, and were found more frequently in children with high CRP and likely pneumococcal pneumonia. Decreased sensitivity for crackles on digital auscultation may be caused by difficulties differentiating artefacts such as stethoscope movement from true lung sounds, especially from a remote recording. However, there were consistently high rates of crackles in all groups for conventional auscultation, including among children likely to have an acute viral infection where crackles may not be as frequently expected (online supplemental table S2), suggesting the potential for false positives on conventional auscultation. Using digital auscultation, crackles were highest in the group most often associated with crackles (pneumococcal pneumonia), less common in likely acute viral infection groups, and rare among controls. Although these patterns may suggest that digital auscultation results in fewer false positives for crackles, without a gold standard measurement, it cannot be ruled out that digital auscultation may be less sensitive for crackles. Nonetheless, presence of crackles-only on digital auscultation may help identify children with higher risk of severe disease and mortality.

There was heterogeneity between the sites in terms of patient and epidemiological characteristics, and with regards to provider level and established training practices on conventional auscultation within and between sites. However, providers conducting conventional auscultation were generally experienced doctors, clinical officers or nurses who regularly conducted clinical assessments for children with pneumonia at each site. Further, there were no significant demographic or clinical predictors of concordance other than The Gambia site being associated with better concordance. The consistency of classifications across several sites with varied severity characteristics suggests that findings may generalise across a wide range of settings.

This evaluation had several limitations. There was no gold standard when comparing conventional and digital auscultation classifications, so there are inferential limits when comparing differences in findings. We are unable to fully evaluate the contribution of multiple sources of variation, including equipment, timing and inter-rater differences. As previously reported, PERCH conducted clinical standardisation trainings and assessments before and during the study.^28^ The course included a brief conventional auscultation training which may have reduced inter-rater differences and provided greater consistency in auscultatory concordance over time and between sites (online supplemental table S5). While the digital auscultation listening panel received a different auscultatory training as part of the listening panel standardisation process, concordance between digital and conventional auscultation may have been improved in general by participation in auscultation training sessions. There was often a time difference between conventional and digital auscultation. While we had an exact time for the digital auscultation recording, our best estimate of the conventional auscultation was the start of the clinical assessment, which could take over an hour. To include all near-simultaneous conventional auscultations and digital recordings, we allowed for a window of 2 hours for concordance evaluations. Longer durations between conventional and digital auscultation were primarily due to availability of staff trained on the digital auscultation process. Nonetheless, concordance was similar when comparing recordings within 2 hours to those recorded within 24 hours (online supplemental table S3). A digital recording review panel may not be available in real-world settings. However, concordance using classifications from a single initial reviewer on the digital auscultation panel was similar to concordance using the panel (online supplemental table S6); the feasibility of having one remote listener is realistic for telemedicine. Alternatively, algorithms may be developed and integrated into digital auscultation systems that provide point-of-care diagnostic information without the need for clinician interpretation. Automated systems could be developed to help identify children at higher risk of severe disease (crackles-only), or conversely, children with wheeze who may benefit from supportive care without antimicrobial therapy.

Conventional and digital auscultation have moderate concordance and are clinically informative; both demonstrate an association between wheeze and decreased clinical severity. Digital stethoscopes may offer value in research where inter-provider variability can be reduced, and in telemedicine, particularly in low-resource settings where the burden of disease is greatest and where trained auscultation may not be available. As viral disease contributes increasingly to paediatric pneumonia, further studies may inform how detection of wheeze on digital auscultation can contribute to case management and offer opportunities for reducing unnecessary antimicrobial use.

## Data Availability

All available data can be obtained by contacting the corresponding author.
