# Supplementary material for: Digitally recorded and remotely classified lung auscultation compared with conventional stethoscope classifications among children aged 1–59 months enrolled in the Pneumonia Etiology Research for Child Health (PERCH) case–control study
Source: BMJ Open Respir Res. 2022 May 16;9(1):e001144. doi: 10.1136/bmjresp-2021-001144 (PMC9115042; doi:10.1136/bmjresp-2021-001144)
Supplement: Supplementary data [file bmjresp-2021-001144supp001.pdf]

Digitally recorded and remotely classified lung auscultation compared with conventional stethoscope classifications among children aged 1–59 months enrolled in the Pneumonia Etiology Research for Child Health (PERCH) case–control study

Contents

Supplemental Table S1. Characteristics of children with severe pneumonia and community controls eligible for the digital auscultation substudy. 2

Supplemental Table 2. Auscultation classifications by digitally recorded and panel classified auscultation and conventional stethoscope auscultation by local provider, by clinical and etiologic categories. 4

2a. Among all digital auscultation substudy cases 4

2b. Among cases that had concurrent digital recording and conventional auscultation (within 2 hours). 5

Supplemental Table 3. Concordance between digitally recorded and classified lung auscultation and conventional stethoscope classifications, by duration between digital recording and conventional auscultation classification. 7

Supplemental Table S4. Case and control status sensitivity and specificity of no crackles or wheeze and any crackles and/or wheeze on digital auscultation classification. 8

Supplemental Table 5. Concordance between digitally recorded and classified lung auscultation and conventional stethoscope classifications, by site among recordings taken within 2 hours of conventional auscultation classification. 9

Supplemental Table 6. Concordance between digitally recorded and classified lung auscultation and conventional stethoscope classifications using the first digital auscultation listener classification (single review), among concurrent digital recordings and conventional auscultation classification. 10

Supplemental Table S1. Characteristics of children with severe pneumonia and community controls eligible for the digital auscultation substudy.

Characteristic	No. (% with available information)				p-value	
	A. Digital auscultation cases	B. Non-enrolled cases	C. Digital auscultation controls	D. Non-enrolled controls	A v B	C v D
**Total**	793	607	301	1463		
**Age (col %)**					**0.005**	**0.018**
1-5m	337 (42.5)	293 (48.3)	73 (24.3)	467 (31.9)		
6-11m	186 (23.5)	156 (25.7)	70 (23.3)	360 (24.6)		
12-23m	166 (20.9)	109 (18.0)	83 (28.6)	354 (24.2)		
24-56m	104 (13.1)	49 (8.1)	75 (24.9)	282 (19.3)		
**Female sex (col %)**	343 (43.3)	250 (41.2)	140 (46.5)	704 (48.2)	0.438	0.604
**Site (row %)**					**<0.001**	**<0.001**
Zambia	252 (88.4)	33 (11.6)	39 (12.0)	387 (88.0)		
Bangladesh	148 (100.0)	0 -	26 (15.8)	139 (84.2)		
Kenya	147 (85.0)	26 (15.0)	6 (1.8)	326 (98.2)		
South Africa	99 (23.6)	320 (76.4)	12 (3.0)	387 (97.0)		
The Gambia	83 (27.0)	224 (73.0)	46 (15.0)	261 (85.0)		
Thailand	64 (94.1)	4 (5.9)	172 (72.9)	64 (27.1)		
**HIV Positive**	59 (7.4)	36 (5.9)	2 (0.7)	74 (5.1)	0.266	**0.001**
**Malnutrition (weight for age)**	174 (23.4)	124 (22.2)	21 (7.0)	134 (9.3)	0.621	0.190

**Very severe pneumonia (WHO definition)**	264 (33.3)	184 (30.3)	-	-	0.237	-
**Microbiologically confirmed pneumonia**	23/759 (3.0)	17/598 (2.8)	-	-	0.839	-
Confirmed pneumococcal pneumonia	6/759 (0.8)	4/598 (0.7)	-	-	0.795	-
Likely pneumococcal pneumonia	58/666 (8.7)	65/534 (12.2)	-	-	**0.049**	-
**Hypoxemia at admission**	267 (33.8)	291 (48.2)	-	-	**<0.001**	-
**Supplemental oxygen (ever)[a]**	355 (44.9)	358 (59.1)	-	-	**<0.001**	-
**Tachypnea**	641 (82.0)	501 (84.3)	-	-	0.246	-
**Malaria parasitemia**	18/776 (2.3)	5/586 (0.9)	1 (0.3)	11 (0.8)	**0.038**	0.408
**Anemia**	21 (2.6)	6 (1.0)	-	-	**0.025**	-
**Abnormal chest X-ray**	333 (44.6)	311 (53.8)	-	-	**0.003**	-
**Death within 30 days of admission**	74/676 (9.3)	39/549 (6.4)	-	-	**0.001**	-
Died in hospital	61/792 (7.7)	35/605 (5.8)	-	-	0.161	-
Died post-discharge, within 30 days of admission	13/676 (1.9)	4/549 (0.7)	-	-	0.076	-
Missing 30 day vital status	117/793 (14.8)	58/607 (9.6)	-	-	**0.004**	-

Supplemental Table S2. Auscultation classifications by digitally recorded and panel classified auscultation and conventional stethoscope auscultation by local provider, by clinical and etiologic categories.

2a. Among all digital auscultation substudy cases

Population and auscultation category	Digital auscultation n (col %)	Conventional auscultation n (col %)	p-value[a]
**Overall cases**	737	737	**<0.001**
No crackles or wheeze	281 (38.1)	214 (29.0)	
Crackles only	90 (12.2)	270 (36.6)	
Any wheeze	366 (49.7)	253 (34.3)	
Wheeze only	167 (22.7)	47 (6.4)	
Crackles and wheeze	199 (27.0)	206 (28.0)	
**Overall controls**	284		
No crackles or wheeze	241 (84.9)	-	
Crackles only	7 (2.5)	-	
Any wheeze	36 (12.7)	-	
Wheeze only	26 (9.2)	-	
Crackles and wheeze	10 (3.5)	-	
**High CRP (>= 40 mg/L)**	146	146	**<0.001**
No crackles or wheeze	59 (40.4)	47 (32.2)	
Crackles only	28 (19.2)	73 (50.0)	
Any wheeze	59 (40.4)	26 (17.8)	
Wheeze only	22 (15.1)	3 (2.1)	
Crackles and wheeze	37 (25.3)	23 (15.8)	
**Likely pneumococcal pneumonia**	53	53	**<0.001**
No crackles or wheeze	20 (37.7)	9 (17.0)	
Crackles only	11 (20.8)	32 (60.4)	
Any wheeze	22 (41.5)	12 (22.6)	
Wheeze only	10 (18.9)	0	
Crackles and wheeze	12 (22.6)	12 (22.6)	
**Discharge alive $\leq$ 2 days with virus detected**[b]	129	129	**<0.001**
No crackles or wheeze	44 (34.1)	29 (22.5)	
Crackles only	10 (7.8)	44 (34.1)	

Any wheeze	75 (58.1)	56 (43.4)	
Wheeze only	38 (29.5)	15 (11.6)	
Crackles and wheeze	37 (28.7)	41 (31.8)	

2b. Among cases that had concurrent digital recording and conventional auscultation (within 2 hours).

Auscultation classification	Digital auscultation	Conventional auscultation	p-value[a]
**Overall cases**	383	383	**<0.001**
No crackles or wheeze	123 (32.1)	84 (21.9)	
Crackles only	45 (11.8)	129 (33.7)	
Any wheeze	215 (56.1)	170 (44.4)	
Wheeze only	99 (25.9)	25 (6.5)	
Crackles and wheeze	116 (30.3)	145 (37.9)	
**Overall controls**	284		
No crackles or wheeze	241 (84.9)	NA	
Crackles only	7 (2.5)	NA	
Any wheeze	36 (12.7)	NA	
Wheeze only	26 (9.2)	NA	
Crackles and wheeze	10 (3.5)	NA	
**High CRP (>= 40 mg/L)**	81	81	**<0.001**
No crackles or wheeze	30 (37.0)	20 (24.7)	
Crackles only	14 (17.3)	42 (51.9)	
Any wheeze	37 (45.7)	19 (23.5)	
Wheeze only	16 (19.8)	2 (2.5)	
Crackles and wheeze	21 (25.9)	17 (21.0)	
**Likely pneumococcal pneumonia**	28	28	**0.011**
No crackles or wheeze	10 (35.7)	4 (14.3)	
Crackles only	6 (21.4)	17 (60.7)	
Any wheeze	12 (42.9)	7 (25.0)	
Wheeze only	5 (17.9)	0	
Crackles and wheeze	7 (25.0)	7 (25.0)	
**Discharge alive ≤ 2 days with virus detected[b]**	74	74	**<0.001**
No crackles or wheeze	20 (27.0)	11 (14.9)	

Crackles only	4 (5.4)	23 (31.1)
Any wheeze	50 (67.6)	40 (54.1)
Wheeze only	25 (33.8)	11 (14.9)
Crackles and wheeze	25 (33.8)	29 (39.2)

a. McNemar-Bowker test among normal, crackles only, and any wheeze.
b. Among children who were discharged alive in less three days with a non-colonizing virus detected on NP-OP PCR (likely acute viral infection).

Supplemental Table S3. Concordance between digitally recorded and classified lung auscultation and conventional stethoscope classifications, by duration between digital recording and conventional auscultation classification.

Time between digital and conventional auscultation	N (% with available information)		Agreement on both conventional and digital		Kappa Coefficient (95% CI)	PABAK Coefficient (95% CI)
	Digital	Conventional	n	%		
**Total**	295	295				
**<1 hours, n=295**						
No crackles or wheeze	83 (28.1)	52 (17.6)	36	78.6%	0.404 (0.286-0.522)	0.568 (0.474-0.661)
Any crackles and/or wheeze	212 (71.9)	243 (82.4)	196			
**<2 hours, n=383**						
No crackles or wheeze	123 (32.1)	84 (21.9)	56	75.2%	0.379 (0.280-0.479)	0.504 (0.413-0.587)
Any crackles and/or wheeze	260 (67.9)	299 (78.1)	232			
**<24 hours, n=649**						
No crackles or wheeze	241 (37.1)	182 (28.0)	121	72.1%	0.371 (0.297-0.445)	0.440 (0.371-0.509)
Any crackles and/or wheeze	408 (62.9)	467 (72.0)	347			

Supplemental Table S4. Case and control status sensitivity and specificity of no crackles or wheeze and any crackles and/or wheeze on digital auscultation classification.

Auscultation classification	Case n (col %)	Control n (col %)	Chi-squared p-value
**All digital auscultation cases and controls**			<0.001
Any crackles and/or wheeze	456 (61.4)*	43 (15.1)	
No crackles or wheeze	281 (38.1)	241 (84.9)**	
**Controls and WHO-defined severe pneumonia cases**			<0.001
Any crackles and/or wheeze	332 (67.3)*	43 (15.1)	
No crackles or wheeze	161 (32.7)	241 (84.9)**	
**Controls and WHO-defined very severe pneumonia cases**			<0.001
Any crackles and/or wheeze	124 (50.8)*	43 (15.1)	
No crackles or wheeze	120 (49.2)	241 (84.9)**	

* Sensitivity; ** Specificity

Supplemental Table S5. Concordance between digitally recorded and classified lung auscultation and conventional stethoscope classifications, by site among recordings taken within 2 hours of conventional auscultation classification.

Comparison	N (% with available information)		Agreement on both conventional and digital		Kappa Coefficient (95% CI)	PABAK Coefficient (95% CI)
	Digital	Conventional	n	%		
**Zambia**	154	154				
No crackles or wheeze	70 (45.4)	58 (37.7)	39	67.5%	0.34 (0.19-0.48)	0.35 (0.20-0.50)
Any crackles and/or wheeze	84 (54.6)	96 (62.3)	65			
**Bangladesh**	121	121				
No crackles or wheeze	27 (22.3)	0 (-)	0	77.7%	0.22 (0.15-0.30)	0.54 (0.39-0.69)
Any crackles and/or wheeze	94 (77.7)	121 (100)	94			
**Kenya**	14	14				
No crackles or wheeze	10 (71.4)	11 (78.6)	10	92.9%	0.81 (0.46-1.00)	0.75 (0.43-1.00)
Any crackles and/or wheeze	4 (28.6)	3 (21.4)	3			
**South Africa**	1	1				
No crackles or wheeze	0	0	0	-	-	-
Any crackles and/or wheeze	1	1	1			
**The Gambia**	71	71				
No crackles or wheeze	8 (11.3)	9 (12.7)	3	84.5%	0.27 (-0.05-0.58)	0.67 (0.49-0.84)
Any crackles and/or wheeze	63 (88.7)	62 (87.3)	57			
**Thailand**	22	22				
No crackles or wheeze	8 (36.4)	6 (27.3)	4	72.7%	0.38 (-0.03-0.78)	0.46 (0.08-0.83)
Any crackles and/or wheeze	14 (63.6)	16 (72.7)	12			

Supplemental Table S6. Concordance between digitally recorded and classified lung auscultation and conventional stethoscope classifications using the first digital auscultation listener classification (single review), among concurrent digital recordings and conventional auscultation classification.

Comparison	N (% with available information)		Agreement on both conventional and digital		Kappa Coefficient (95% CI)	PABAK Coefficient (95% CI)
	Digital	Conventional	n	%		
**Total**	377	377				
**Auscultation Classification**						
No crackles or wheeze	130 (34.5)	80 (21.2)	56	74.0%	0.367 (0.269-0.465)	0.481 (0.393-0.570)
Any crackles and/or wheeze	247 (65.5)	297 (78.8)	223			
